# Structural Insights into Seeding Mechanisms of hIAPP Fibril Formation

**DOI:** 10.1021/jacs.3c14233

**Published:** 2024-05-09

**Authors:** Saba Suladze, Christian Sustay Martinez, Diana C. Rodriguez Camargo, Jonas Engler, Natalia Rodina, Riddhiman Sarkar, Martin Zacharias, Bernd Reif

**Affiliations:** 1 Bayerisches NMR Zentrum (BNMRZ) at the Department of Biosciences, School of Natural Sciences, Technische Universität München, 85747 Garching, Germany; 2 Helmholtz-Zentrum München (HMGU), Deutsches Forschungszentrum für Gesundheit und Umwelt, Institute of Structural Biology (STB), Ingolstädter Landstraße 1, 85764 Neuherberg, Germany; 3 Center for Functional Protein Assemblies (CPA), Department of Bioscience, TUM School of Natural Sciences, Technische Universität München, Ernst-Otto-Fischer-Straße 8, 85747 Garching, Germany

## Abstract

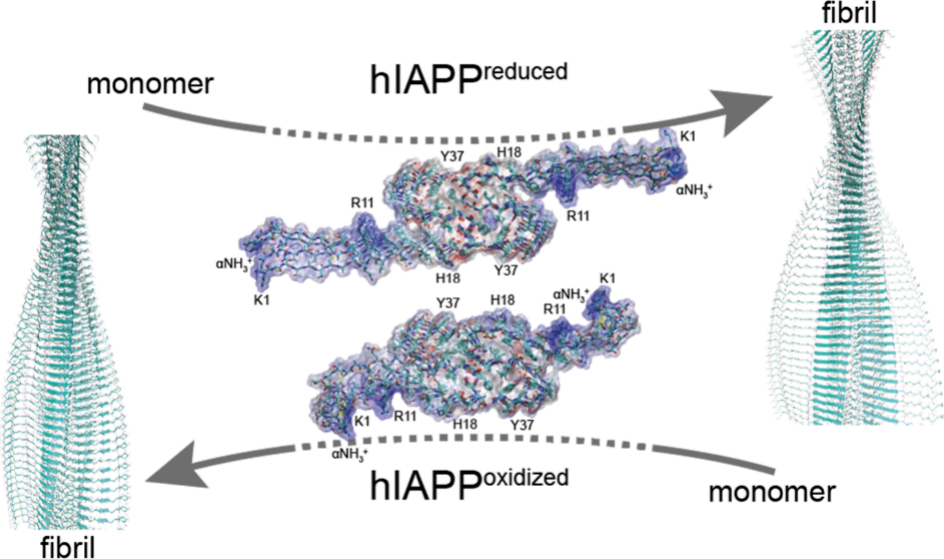

The deposition of islet amyloid polypeptide (hIAPP) fibrils is a hallmark of β-cell death in type II diabetes. In this study, we employ state-of-the-art MAS solid-state spectroscopy to investigate the previously elusive N-terminal region of hIAPP fibrils, uncovering both rigidity and heterogeneity. Comparative analysis between wild-type hIAPP and a disulfide-deficient variant (hIAPP_C2S,C7S_) unveils shared fibril core structures yet strikingly distinct dynamics in the N-terminus. Specifically, the variant fibrils exhibit extended β-strand conformations, facilitating surface nucleation. Moreover, our findings illuminate the pivotal roles of specific residues in modulating secondary nucleation rates. These results deepen our understanding of hIAPP fibril assembly and provide critical insights into the molecular mechanisms underpinning type II diabetes, holding promise for future therapeutic strategies.

## Introduction

Our understanding of amyloid fibril structures has dramatically improved in recent years driven by developments in cryo-EM, solid-state NMR, and X-ray fiber diffraction yielding atomic-resolution structures of amyloid fibrils formed *in vitro* and extracted from patient tissues.^1^ While the rigid cross-β spine parts that constitute the amyloid core are well understood, the characterization of peripheral relatively flexible regions, which are often crucial for specific biological functions, remains an open challenge.^2,3^

In this work, we focus on the neuroendocrine peptide hormone human islet amyloid polypeptide (hIAPP) that is found to be aggregated in amyloidogenic plaques in individuals suffering from type 2 diabetes (T2DM).^4^ Under conditions of hyperglycemia, the secretion of insulin and hIAPP are upregulated with a disproportionate increase of hIAPP.^5^ Increased secretion of hIAPP in severe hyperglycemia has been suggested to facilitate the formation of hIAPP derived amyloid deposits and pancreatic islet β-cell dysfunction.^6^

In recent cryo-EM hIAPP fibril structures,^7−9^ a significant amount of the hIAPP sequence (residues 1–12) is not resolved in the electron density maps, presumably because of extensive polymorphism. Alternatively, the N-terminus of hIAPP is considered to be dynamic and flexible.^10,11^ The relevance of the semirigid solvent-facing residues outside the fibril core has been demonstrated for different amyloid-forming proteins.^12−18^ These regions have been shown to play key parts in the interaction of amyloid fibrils with other cellular components such as RNA molecules,^19^ cell membrane binding/disruption,^20^ and molecular chaperones.^21^ Furthermore, terminal domains of amyloidogenic proteins have been reported to modulate the aggregation mechanism and toxicity.^2,22−25^ hIAPP fibril assembly is largely controlled by the disulfide bond involving Cys2 and Cys7 at the N-terminus.^26^ We have previously shown that the oxidation state of the N-terminal domain defines the stability of the native state and consequently has an effect on the hIAPP fibril formation kinetics.^27^ At the same time, the hIAPP N-terminal domain influences amyloid fibril assembly by interfering with both primary and secondary nucleation in the fibril growth kinetics.^26^ The disulfide bridge is present in all species (including human, cat, rat, and monkey) and is required for IAPP’s biological activity.^28^ Considering both the physiological and biophysical importance of the disulfide bond in hIAPP, better insight into the conformational properties of the N-terminal region is crucial for a deeper understanding of hIAPP aggregation and pathology.

In this work, we examine the role of the N-terminal disulfide in the assembly of hIAPP fibrils. We report perturbations in the assembly kinetics of hIAPP fibrils upon removal of the disulfide bond. In addition, we performed proton-detected MAS solid-state NMR experiments of ^2^H, ^13^C, ^15^N isotopically labeled hIAPP fibrils. The hIAPP peptide was prepared recombinantly and was amidated at the C-terminus.^29^ We obtained almost complete site-specific resonance assignments and performed a chemical shift analysis to yield secondary structure information. Furthermore, we acquired long-range ^1^H,^1^H distance restraints and calculated a structural model for hIAPP fibrils. We compare the obtained model with the published cryo-EM structures and find that the N-terminus is well-defined and homogeneous, irrespective of the hIAPP redox state. The removal of the disulfide bonds results in the formation of an extended N-terminal β-sheet structure, which increases the amount of intermolecular hydrogen bonding in the fibril state. To further characterize structural differences in the wt hIAPP and hIAPP_C2S,C7S_ N-terminal region within the fibrils, we performed molecular dyanmics (MD) simulations.

## Experimental Section

### Recombinant Expression and Fibrillation of hIAPP

Full-length hIAPP (KCNTATCATQRLANFLVHSSNNFGAILSSTNVGSNTY-NH_2_), both unlabeled and uniformly, isotopically labeled, was prepared as described previously.^29^ This protocol allows production of wt hIAPP peptide, which is amidated at the C-terminus and which contains a disulfide bridge involving residues Cys-2 and Cys-7. Briefly, hIAPP is expressed in *E. coli* as a fusion protein with an N-terminal solubility tag, and a C-terminal intein-chitin binding domain (CBD) affinity tag. The C-terminal tag is cleaved off by making use of an intein splicing reaction by incubation of the protein in a solution containing ammonium bicarbonate, which yields the C-terminal amide of native hIAPP. The N-terminal solubility tag is subsequently cleaved using V8 protease. The cleavage products are separated by affinity chromatography, filtration, and reverse phase-HPLC. Finally, the isolated peptide is treated with H_2_O_2_ in acetate buffer to create the disulfide bond. Purified hIAPP was lyophilized and stored at −80 °C until further use. Uniformly ^2^H,^13^C,^15^N-labeled hIAPP was expressed in deuterated (∼99.8% D) M9 medium supplemented with ^15^NH_4_Cl and ^2^H,^13^C-glucose. Under these conditions, we obtained a yield of ∼1.5 mg of purified peptide per liter of culture. The expression and purification of hIAPP_C2S,C7S_ peptide was accomplished following the same procedures (without incubation in H_2_O_2_). QuikChange site-directed mutagenesis was performed to produce the hIAPP_C2S,C7S_ plasmid by using the PfuTurbo DNA polymerase standard protocol (mutagenesis kit, Agilent Technologies) and a temperature cycler. The correctness of the clone was confirmed using DNA sequencing (GACT Biotech). Molecular biology reagents were obtained from Roche, Sigma-Aldrich St. Louis, MO, USA and New England Biolabs. Isotopically labeled nutrients were purchased from Cambridge Isotope Laboratories (CIL).

Fibrillation was achieved by dissolving lyophilized peptide powder to a final concentration of 1 mg/mL peptide with 30 mM acetic acid buffer (pH 5.3, 0.02% NaN_3_) and incubation of the peptide solution for 30 days in a shaker (New Brunswick Innova 40) at 150 rpm at 37 °C in a 5 mm NMR glass tube (Bruker) placed horizontally. *Ex vivo* seeds extracted from pancreatic islets of hIAPP transgenic mice were obtained as described in detail by Franko et al.^30^ These seeds were used in seeding reactions (10% w/w) to produce seeded hIAPP fibrils. After fibrillation, the highly viscous sample was filled into a 1.3 mm Bruker MAS rotor (1.7 μL sample volume) by ultracentrifugation. The pellet was sealed with custom-made spacers to prevent the sample from dehydration.

### Thioflavin-T Assay

Amyloid aggregation kinetics was monitored by the amyloid-specific dye Thioflavin-T (ThT). Peptide dissolved in 20 mM phosphate (pH 7.4) was incubated at 25 °C using a 96-well plate (SpectraMax iD5, Molecular Devices) at concentrations in the range 7.5–25 μM in the presence of 10 μM ThT dye. After excitation at 440 nm, the ThT emission spectra were monitored at 480 nm as a function of time at 10 min intervals. Double-orbital shaking was applied to 96-well plates before each data set collection. The kinetics of the seeded fibril growth was monitored after adding 1–20% w/w fibril seeds to 10 μM peptide under otherwise identical conditions.

### Transmission Electron Microscopy (TEM)

Formvar carbon-coated copper grids 300-mesh (Ted Pella, Inc.) were glow-discharged for 30 s before applying the samples. Five μL of the sample were incubated on the grid for 1.5 min then blotted off with filter paper. The grid was washed two times with a few drops of distilled water. The remaining liquid was wicked off, and then 5 μL of 2% uranyl acetate was applied to the grid. The staining solution was removed by filter paper after an incubation time of 30s. The grid was allowed to dry for 10–15 min. Micrographs were taken with a Ruby camera installed in a JEOL 1400 plus microscope (JEOL) operated at 120 kV at a nominal magnification of 60 k, which resulted in a pixel size of 0.275 nm/px. The scale bar was applied by using ImageJ2 software.

### NMR Spectroscopy

All NMR experiments were performed at 4 °C (keeping a VT gas flow of 1300 L/h), employing a Bruker Avance III 800 MHz solid-state NMR spectrometer, equipped with a triple resonance 1.3 mm MAS probe. In all ^1^H-detected experiments, the MAS rotation frequency was adjusted to 55 kHz (±10 Hz). In the CP-based HSQC experiment, the 90° pulses were set to 1.5 μs by using a ^1^H RF field of 166.6 kHz. For ^15^N, an rf-field amplitude of 46.7 kHz was employed corresponding to a 90° pulse length of 5.35 μs. ^1^H,^15^N CP was achieved using a contact time of 0.8 ms. sl-TPPM^31^ was employed for ^1^H decoupling during ^13^C and ^15^N evolution with ω_RF_/(2π) = 13.75 kHz. WALTZ-16^32^ was applied for ^15^N and ^13^C decoupling during ^1^H acquisition (ω_RF_/(2π) = 10 kHz). In addition, for 3D assignment experiments, ^13^C 90° pulses of 3.4 μs (ω_RF_/(2π) = 73.53 kHz) were employed. For both the CO-N and the Cα-N CP step, optimal control derived tmSPICE pulse schemes (ω_RF_/(2π) = 40 kHz on ^13^C and ^15^N) were applied, scaled accordingly to match the MAS frequency.^33,34^ CO-Cα or Cα-Cβ transfers were achieved using out-and-back scalar transfers suggested by Barbet-Massin.^35^ MISSISSIPPI was applied for 300 ms to achieve water suppression.^36^ Quadrature detection in all indirect dimensions was achieved by employing States-TPPI. To determine the fibril fold, a 3D hNh-RFDR-hNH correlation experiment^37,38^ was recorded to probe ^1^H–^1^H proximities. ^1^H–^1^H RFDR mixing was achieved employing π pulses with ω_RF_/(2π) = 100 kHz for 6 ms. XY-4 phase cycle was employed during the RFDR pulse train. The rest of the acquisition parameters were kept the same as those above. *t*_aq_= 10 ms was set for the two ^15^N indirect dimensions with a spectral width of 30 ppm, respectively. NMR spectra were processed using the software TopSpin (Bruker) and analyzed using ccpNMR analysis.^39,40^ Overall, the completeness of assignment amounted to 92.4% and 87.5% for wt hIAPP and hIAPP_C2S,C7S_, respectively. The proton chemical shifts were referenced to the water resonance frequency, and the ^15^N and ^13^C shifts were referenced indirectly. The NMR chemical shifts of wt hIAPP and hIAPP_C2S,C7S_ fibrils are deposited in the BMRB (accession codes 51865 and 51866).

### CYANA Structure Calculations

Fibril structure calculations followed the protocol established for solid-state NMR with CYANA.^41−43^ Structure calculations were performed for a fibril comprising five identical layers spaced ∼4.7 Å apart with one molecule per layer. Five monomers were held in identical conformation by introducing an identity restraint function limiting dihedral angle differences between identical residues on different subunits.^41^ The five parallel β-sheets were restrained by introducing hydrogen bonds (up or down along the fibril axis) using upper and lower distance bounds of 1.8 ≤ *d*_OH_ ≤ 2.0 Å and 2.7 ≤ *d*_ON_ ≤ 3.0 Å.^44^ Additionally, β-sheet formation was supported by the use of backbone torsion angles (for residues involved in a β-sheet) as generic restraints in the ranges −170° ≤ φ ≤ −110° and 110° ≤ ψ ≤ 170°. Prior to structure calculation, 30 intramolecular medium- and long-range distance restraints (listed in Supporting Information, Table S4), identified in the 6 ms 3D hNh- RFDR-hNH correlation experiment, were introduced for each subunit. In addition, the disulfide bond between residues Cys-2 and Cys-7 was imposed as a distance restraint in the structure calculation. With this set of restraints, a manual 3D structure was calculated using upper distance bounds of 12 Å and lower distance bounds of 9 Å for backbone protons for residues involved in β-sheets. Restraints involving loop and turn residues were allowed to have a lower distance bound of 7 Å. This procedure resulted in a well-converged structure with an average target function of 2 Å^2^ for the final bundle, comprising the 10 best conformers, with an average heavy atom root mean square deviation (RMSD) of 1.9 Å, and an average backbone RMSD of 1.5 Å. The quality of the final structural ensemble of wt hIAPP fibrils was evaluated with PROCHECK^45^ and PSVS.^46^ The restraints and structure statistics are given in Table S5.

### Modeling

To gain more insight into the mechanism that underlies fibril seeding, we performed molecular dynamics simulations. The starting model was prepared by attaching residues 1–12 to the cryo-EM structure^7^ (PDB code 6Y1A). The variant hIAPP_C2S,C7S_ was modeled using cysteines in the reduced state. For both peptides, amidation at the C-terminus was assumed in the simulations. In the calculations, 4.7 Å distance restraints were applied between hydrogen-bond donors and acceptors for residues that are known to adopt β-sheet structure from the NMR experiments (residues 8–12 and 14–20 for wt hIAPP, and residues 3–11 and 15–20 for hIAPP_C2S/C7S_). The force constant of these restraints was set to 20 kcal/(mol·Å^2^). In both cases, an 8-layer amyloid was simulated for 1 μs using the ff19SB force field,^47^ employing an OPC water model,^48^ applying the Langevin thermostat,^49^ and the Berendsen barostat,^50^ along with a 10 Å cutoff for direct space nonbonded interactions. All simulations were carried out using the AMBER22 suite.^51^ β-Sheet propensities obtained from the simulations were calculated using the DSSP method^52^ from MDTraj^53^ (version 1.9.7) on the inner 4 layers of the simulated amyloid in order to decrease boundary effects. Similarly, an estimated order parameter S2 was obtained from the chain conformations sampled from the MD trajectory using pytraj.^54^ The distance between the centers of mass of the side chains of residues K1 and R11 was calculated to yield a distance distribution using the Kernel Density Estimation (KDE) implementation of scikit-learn (version 1.2.1).^55^ For representative structures the electrostatic potential was calculated using the adaptive Poisson–Boltzmann solver (APBS) method.^56^

## Results and Discussion

**Figure 1:**
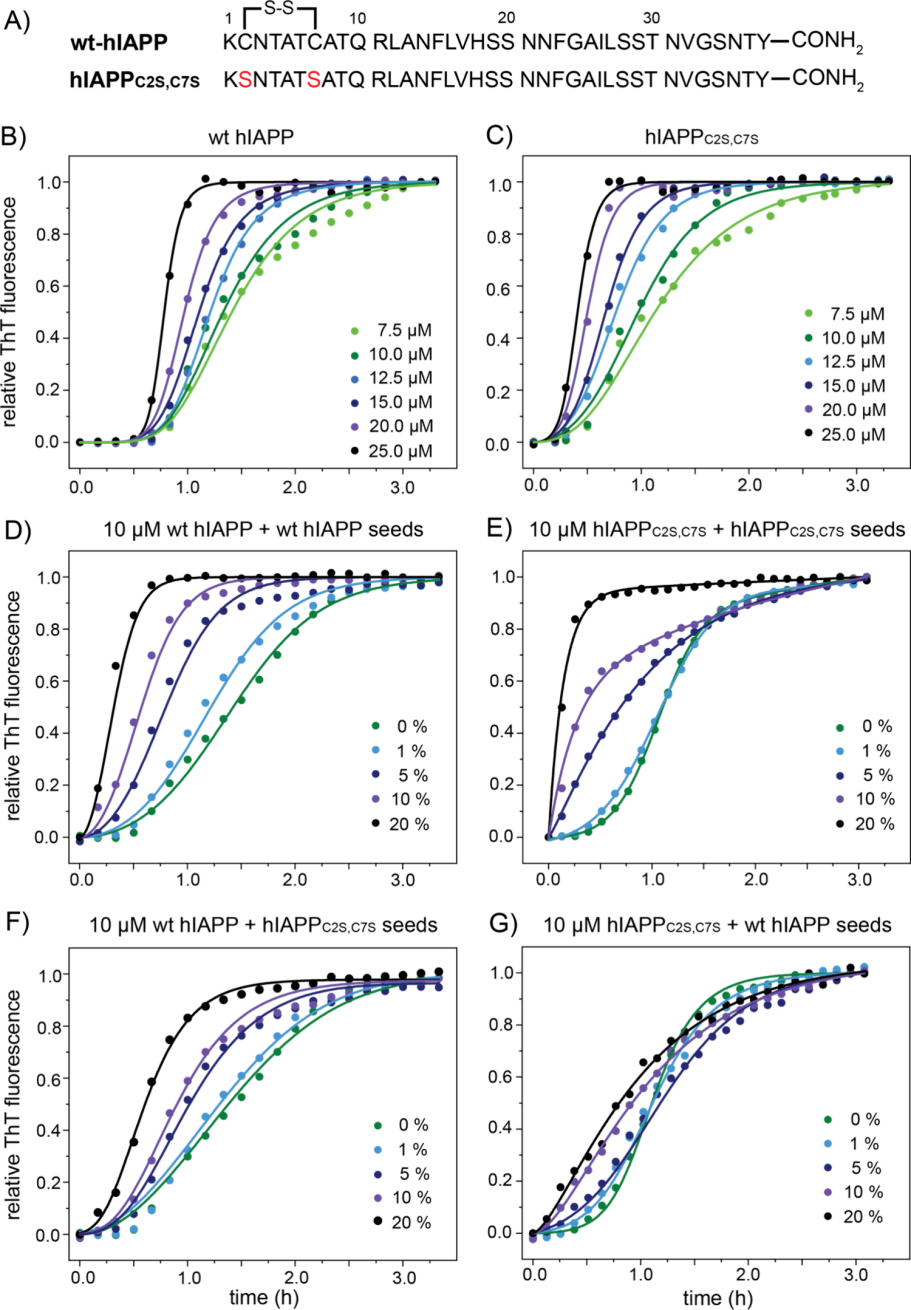
(A) Amino acid sequences of wt hIAPP and variant hIAPP_C2S,C7S_. Both peptides are amidated at the C-terminus. (B, C) Concentration-dependent aggregation kinetics of wt hIAPP (left) and hIAPP_C2S/C7S_ (right). The normalized ThT fluorescence intensity of wt hIAPP and hIAPP_C2S,C7S_ is plotted as a function of time. In the experiment, the concentration was varied in the range of 7.5–25 μM. The experimental data are depicted as circles. Lines represent the best global fit to the secondary nucleation dominated model. Fitted values for combined rate constants for primary *k*_n_·*k*_+_ and secondary *k*_2_·*k*_+_ processes are shown in Table S1. (D, E) Seeded growth kinetics for wt hIAPP (left) and the variant hIAPP_C2S,C7S_ (right). 0–20% (v/v) of seeds have been added to a 10 μM peptide solution. (F, G) Cross-seeding of hIAPP. On the left, hIAPP_C2S/C7S_ seeds are added to monomeric wt-hIAPP, while wt-hIAPP seeds are titrated to monomeric hIAPP_C2S/C7S_ on the right. The concentration of seeds was varied in the range 0–20% (v/v).

Figure 1B,C shows the kinetics of amyloid formation of wt hIAPP and the hIAPP_C2S,C7S_ variant monitored by the amyloid-sensitive fluorescent dye thioflavin T (ThT). The aggregation assay was performed in a concentration-dependent manner in the concentration range of 7.5–25 μM. The aggregation kinetics for both peptides is highly reproducible, resulting in a sigmoidal growth behavior comprising a lag phase, an exponential growth phase and the final plateau, which is typical for nucleation-dependent fibril growth.^57^ The aggregation half time, i.e., the time needed to reach the half-maximum ThT fluorescence intensity, decreases with peptide concentration for both peptides. At all concentrations tested, hIAPP_C2S,C7S_ aggregates faster than wt hIAPP. This is consistent with a previous report where it was shown that reduced hIAPP has a higher aggregation propensity.^27^ To gain further insight into the mechanism of hIAPP fibril formation, we carried out a global kinetic analysis of hIAPP aggregation using the fitting tool AmyloFit (http://www.amylofit.ch.cam.ac.uk).^58^ This platform allows dissecting of the aggregation process to extract rate constants for the individual microscopic steps. In particular, the rates *k*_+_, *k*_n_, and *k*_2_ can be determined which refer to the elongation rate at the fibril ends, the rate of primary nucleation in solution, and the secondary nucleation rate on the fibril surface, respectively. Global fitting shows that hIAPP aggregation is best described by a secondary nucleation dominated model, in agreement with previously published data.^26,59,60^ This suggests that the majority of new aggregates are formed via surface-catalyzed secondary nucleation rather than primary nucleation. While elongation and secondary nucleation rates are comparable for wt hIAPP and hIAPP_C2S,C7S_, the primary nucleation rate *k*_n_ of the disulfide-free variant is 100-fold greater than that for wt hIAPP (Supporting Information Table S1), consistent with a shorter lag phase and faster assembly into amyloid. While the N-terminal disulfide does not contribute to the amyloid fiber core, it still plays a central role in the assembly mechanism. Removal of the disulfide results in reduced helical propensity for the N-terminal residues 8–18,^27^ which might interfere with primary nucleation. In addition, the disulfide bond may play a protective role against amyloid formation by reducing the number of potential intermolecular hydrogen bonding interactions which in turn could promote oligomerization.^61^

Next, we examined the kinetics of seeded fibril growth for each of the hIAPP peptides. Seeded fibril growth was initiated by adding 0–20% fibril seeds at the beginning of the aggregation reaction. As expected, the lag time for both wt hIAPP and hIAPP_C2S,C7S_ aggregation is significantly reduced, even in the presence of low concentrations of fibril seeds, indicating that secondary nucleation is important for both peptides. When the seeded aggregation kinetics of the wt hIAPP and hIAPP_C2S,C7S_ are compared, we find that the disulfide-free double mutant hIAPP_C2S,C7S_ forms amyloids faster (Figure 1D,E). The loss of the disulfide eliminates the sigmoidal nature of the seeded kinetics observed for wt hIAPP. Apparently, an activation step is required for the oxidized peptide, implying the addition of oligomers or a conformational conversion during secondary nucleation. This is consistent with results that have been reported previously by Miranker and co-workers.^26^ For both wt hIAPP and hIAPP_C2S,C7S_ seeded growth, the slope of the ThT growth curves increase with seed concentration, suggesting that elongation, i.e., addition of monomeric protein molecules onto seed fibril ends, contributes significantly to the aggregation kinetics.^62,63^

In addition, we performed cross-seeding experiments (Figure 1F,G). While hIAPP_C2S,C7S_ seeds are still able to catalyze fibril formation of wt hIAPP, the use of wt hIAPP seeds dramatically slows down fibril formation of hIAPP_C2S,C7S_. Seeding of wt hIAPP with hIAPP_C2S,C7S_ seeds is almost as efficient as self-seeding with wt hIAPP fibrils, while seeding of hIAPP_C2S,C7S_ using wt hIAPP seeds compares nearly to the nonseeded fibril growth curves. The ability of hIAPP_C2S,C7S_ seeds to cross-seed fiber formation of soluble wt hIAPP is presumably due to the structural similarities of both fibril structures. This is in agreement with a study which suggests that the aromatic residues F23 and Y37 are structurally indistinguishable in both fibril structures.^26^ At the same time, removal of the disulfide does not induce any detectable effect on hIAPP toxicity *in vitro* suggesting that oligomers formed by these two polypeptides share common structural features.^20^

### Assignment of the NMR Chemical Shifts of hIAPP Fibrils

**Figure 2:**
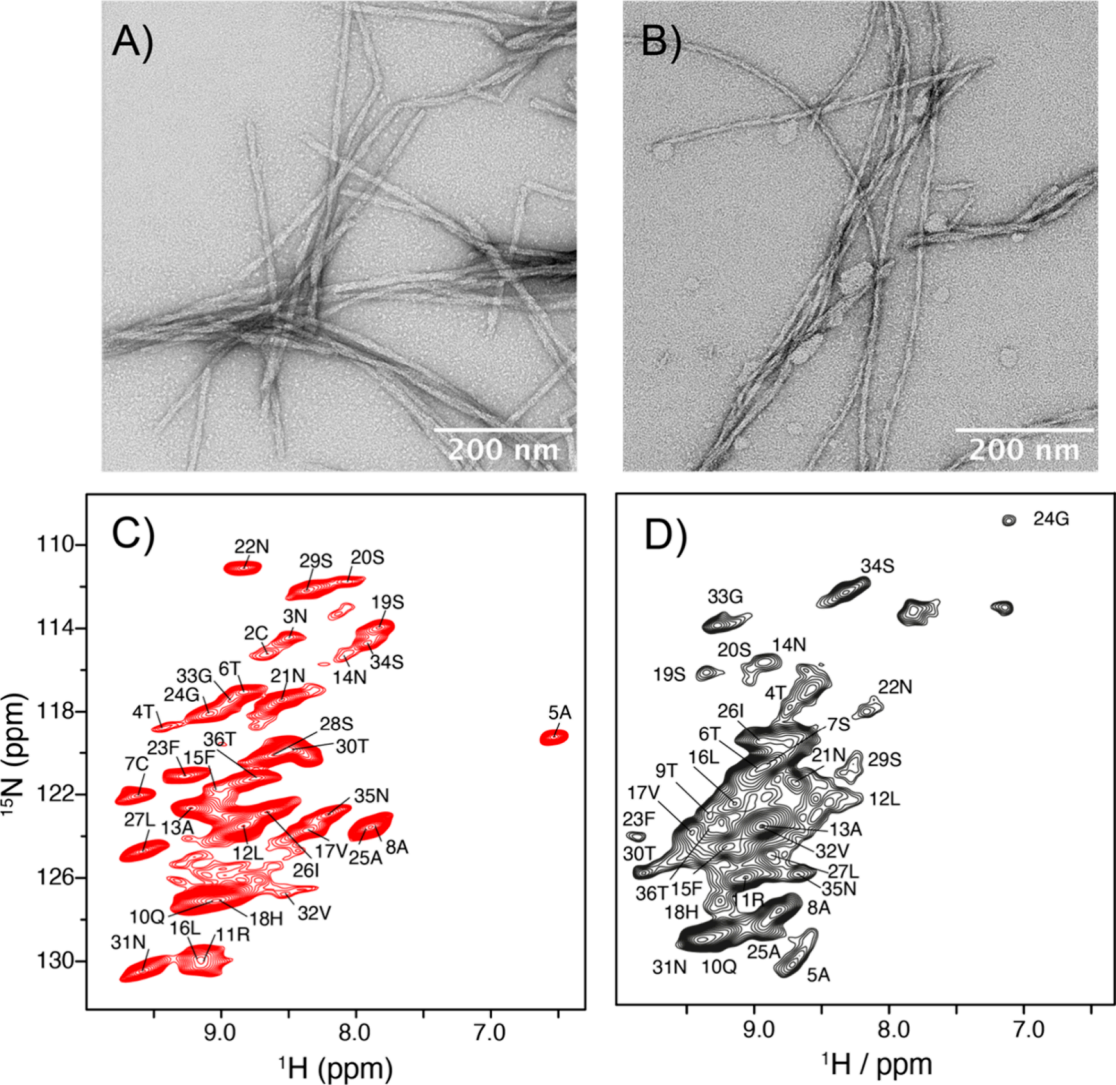
Representative TEM images of wt-hIAPP (A) and IAPP_C2S,C7S_ fibrils (B) employed for the NMR spectroscopic experiments. MAS solid-state NMR ^1^H,^15^N correlation spectra of wt-hIAPP (C) and IAPP_C2S,C7S_ fibrils (D) with assignments. NMR experiments were recorded using perdeuterated fibril samples in which exchangeable sites are back-substituted with protons.

Fibrils were prepared by incubation for 30 days without seeding. Representative 2D ^1^H,^15^N correlation spectra of hIAPP and hIAPP_C2S,C7S_ fibrils are shown in Figure 2C,D. ^1^H^N^ and ^15^N have line widths on the order of 125 and 50 Hz, respectively, and are comparable to the line widths observed in other amyloid fibril samples investigated previously using proton-detected MAS NMR experiments.^64,65^ Even though the ^1^H,^15^N correlation spectra appear to have some spectral overlap, the 3D assignment experiments yield well-defined correlation peaks which are well dispersed (Figure S1). Similar to the oxidized fibrils, the disulfide free hIAPP_C2S,C7S_ fibrils are rigid which can be appreciated by the CP spectrum shown in Figure 2D. At first sight, the hIAPP_C2S,C7S_ fibril sample yields spectra of lower resolution in comparison to the oxidized hIAPP fibril sample. This is presumably because this peptide aggregates faster, which might result in a larger degree of conformational heterogeneity. Interestingly, solid-state NMR samples that are prepared using *ex vivo seeds* extracted from pancreatic islets of hIAPP transgenic mice^30^ yield the same fibril morphology (Figure S2).

Sequential assignments for the perdeuterated and back-substituted ^13^C,^15^N labeled hIAPP fibril samples were obtained using dipolar-based proton-detected 3D hCANH/hcoCAcoNH experiments in combination with 3D hcaCBcaNH and hCONH experiments^35^ (Figure S1). The assignment is based on two 3D chemical shift correlation experiments in which a particular backbone NH pair for residue CA_*i*_ is correlated to the neighboring CA_*i*–1_ chemical shift, yielding a walk through all backbone resonances of the peptide. Amino acid types were identified by making use of their characteristic Cβ chemical shifts. The NMR chemical shifts of wt hIAPP and hIAPP_C2S,C7S_ fibrils are deposited in the BMRB under the accession codes 51865 and 51866, respectively (Tables S2 and S3). We were able to assign all residues except Y37 for the wt hIAPP fibrils. Lys-1 and Thr-9 were partially assigned. For hIAPP_C2S,C7S_ fibrils, all residues could be assigned with the exception of Lys-1, Ser-2, and Tyr-37. Asn-3 was partially assigned. No peak doubling was observed for any of the assigned residues, suggesting that the hIAPP and hIAPP_C2S,C7S_ fibril samples are monomorphic and not heterogeneous. Disulfide bond formation in wt hIAPP fibrils was confirmed by the Cβ chemical shift of Cys-7 which adopts a value of 48.8 ppm. As a consequence of disulfide loop formation, the chemical shifts for Ala-5 differ significantly from the reference random coil chemical shift. Ala-5 yields a Cβ shift of 26.4 ppm and a H^N^ shift of 6.5 ppm. This unusual Cβ chemical shift was also observed previously in ^13^C-detected solid-state NMR experiments.^66,67^

We were able to assign the N-terminal residues of the peptide (residues 1–12), which have been reported to be largely disordered and dynamic.^7,9,68,69^ All of the sequential assignment experiments rely on dipolar coupling mediated polarization transfers and are therefore probing regions of the molecule that have a high molecular order and low mobility. Cross peaks for Tyr-37 are presumably not observed because of line broadening induced by static disorder or dynamics for this residue inside the fibril.

### Chemical Shift Analysis

**Figure 3:**
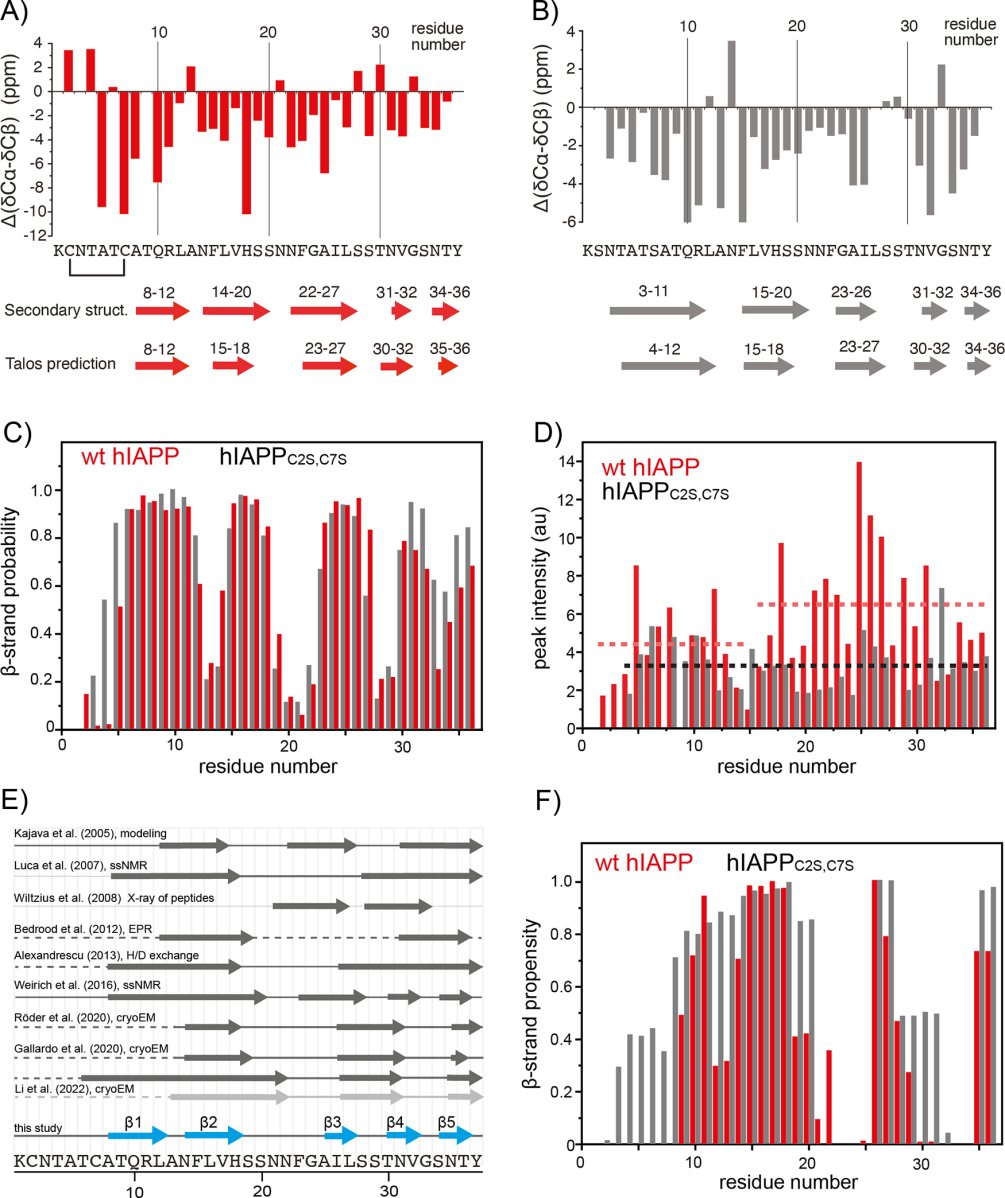
Secondary chemical shift analysis of the oxidized hIAPP fibril (A) and the disulfide-free variant hIAPP_C2S,C7S_ (B). The positions of the β-strands are estimated on the basis of the secondary chemical shift differences. In addition, TALOS-N^55^ predictions are shown. The TALOS analysis is based on the NMR chemical shift values of ^1^^3^Cα, ^1^^3^Cβ, ^1^^3^C′, ^15^N, and ^1^H^N^. Chemical shifts are corrected for deuterium isotopic effects.^56^ (C) β-Sheet propensity for wt hIAPP (red) and IAPP_C2S,C7S_ (gray) fibrils according to TALOS-N. The first β-strand is elongated in the very N-terminal part of the hIAPP_C2S,C7S_ fibril sample. (D) 3D hCANH cross-peak intensity for wt hIAPP (red) and variant IAPP_C2S,C7S_ (gray) fibrils. A reduction in the signal intensity indicates increased residue-specific heterogeneity or dynamics. The dashed lines represent average intensities for specific regions. (E) Schematic representation of hIAPP fibril secondary structure elements identified in previous investigations. Gray arrows and thick lines represent β-sheet structures and defined ordered regions, respectively. Thin lines indicate that the respective residues are not accounted for in the study. Dashed lines refer to residues that are not observable in the reported experiments. Blue arrows indicate the β-sheets found in this study (CYANA calculation). (F) β-Sheet propensity of wt hIAPP and variant IAPP_C2S,C7S_ fibrils obtained from MD simulations.

The sequential assignments directly yield the secondary structure elements of the peptide via analysis of the secondary chemical shifts (ΔδCα and ΔδCβ), i.e., the difference between the experimental ^13^Cα /^13^Cβ chemical shifts and the corresponding random coil ^13^Cα/^13^Cβ chemical shift values.^70^ Three or more adjacent negative values with a chemical shift difference of more than −2 ppm indicate an extended conformation typical of a β-sheet (Figure 3A,B). Alternatively, the chemical shift of assigned residues is analyzed using TALOS-N^71^ to yield predictions for the backbone torsional angles φ and Ψ (Figure 3C). For this purpose, the experimental NMR chemical shifts have been corrected for the deuterium isotopic effect.^72^

For wt hIAPP fibrils, we have identified five β-sheets involving residues 8–12 (β1), 15–18 (β2), 25–27 (β3), 30–32 (β4), and 34–36 (β5). Backbone torsional angle predictions from TALOS-N were classified as “*strong*” for all β-strand regions and “*ambiguous*” at the kink positions for residues A13, S19, N21, N22, S28, and G33. When the secondary structure propensities of wt hIAPP and hIAPP_C2S,C7S_ fibrils are compared, we observe a remarkable similarity, especially for residues 13–37. This implies that the two fibrils share the same cross-β core structure. This information is employed in modeling the wt hIAPP and hIAPP_C2S,C7S_ fibril structures (see below). A correlation of the Cα chemical shifts of wt hIAPP and hIAPP_C2S,C7S_ fibrils is show in Figure S3. The hCANH spectrum for hIAPP_C2S,C7S_ fibrils lacks resonances originating from residues 1–3. At the same time, the hIAPP fibrils without the disulfide bond yield a longer β-strand at the N-terminus involving residues 4–12. The length of the N-terminal β-strand in wt hIAPP fibrils seems shorter since β-strand formation is presumably hindered by the presence of the disulfide bond. The first seven residues of wt hIAPP fibrils are thought to be outside of the structured amyloid core due to steric constraints imposed by the disulfide bridge on the peptide backbone.^73^ This part of the peptide is considered to be heterogeneous or dynamic in its fibril structure. To address this issue, we compared the cross-peak intensities from the 3D hCANH experiment (Figure 3D). Cross-polarization is a dipolar recoupling sequence. The experimental cross-peak intensities are thus directly related to the mobility of a given residue. The majority of residues produce strong cross-peaks, indicating high order parameters and thus a rigid conformation. For wt hIAPP, residues 16–36 show more intense peaks, suggesting that these residues constitute the rigid core of the fibril, while the first 15 residues have lower intensities, implying a higher flexibility or an increased conformational heterogeneity of the N-terminus. Similarly, less intense cross-peaks appear for residues that are part or in the vicinity of loops and turns, such as C2, N3, T4, N14, F15, V32, and G33.

The molecular structure of fibrillar hIAPP is being studied extensively, and various models exist to date (Figure 3E). Solid-state NMR studies,^66,67^ hydrogen–deuterium (H/D) exchange,^74^ and X-ray diffraction of microcrystals of small peptide fragments^75^ were used to yield the localization of the β-sheets in the hIAPP amyloid fibril structure. The consensus from these studies is that hIAPP adopts a U-shaped hairpin structure composed of two symmetrically related β-strand segments connected by a turn. While the hIAPP fibril models obtained from these different techniques vary in the position and length of the C-terminal β-strand (residues 23–37), they agree well for the N-terminal β-sheet (residues ∼8–18). Our data are in agreement with these previous results. Figure 3F shows the β-sheet propensity for wt hIPPA and hIAPP_C2S,C7S_ fibrils obtained from MD simulations.

It is striking, however, that all cryo-EM structures published to date^7−9,76^ are missing ordered residues in the N-terminal region of the peptide involving residues 1–12. A potential β-strand (residues 5–11) corresponding to an ill-defined cryo-EM electron density surrounding the core was suggested for a N-terminal SUMO-tagged and nonamidated hIAPP fibril sample.^76^ In this case, the relatively flexible N-terminus of hIAPP would adopt a preferred conformation, which is at ∼90° to the rest of the protein and is detached from the fibril core. In a very recent study that was carried out in the absence of salt and under conditions of 2% HFIP, a hIAPP fibril structure is obtained in which some parts of the N-terminus of the peptide could be refined in the electron densities.^77^ The basic unit is built from an asymmetric dimer, which would result in two sets of NMR resonances. We assume that under conditions of low salt and in the presence of HFIP a different polymorph is obtained that is not consistent with our solid-state NMR results.

### Modeling of the 3D hIAPP Fibril Structure

**Figure 4:**
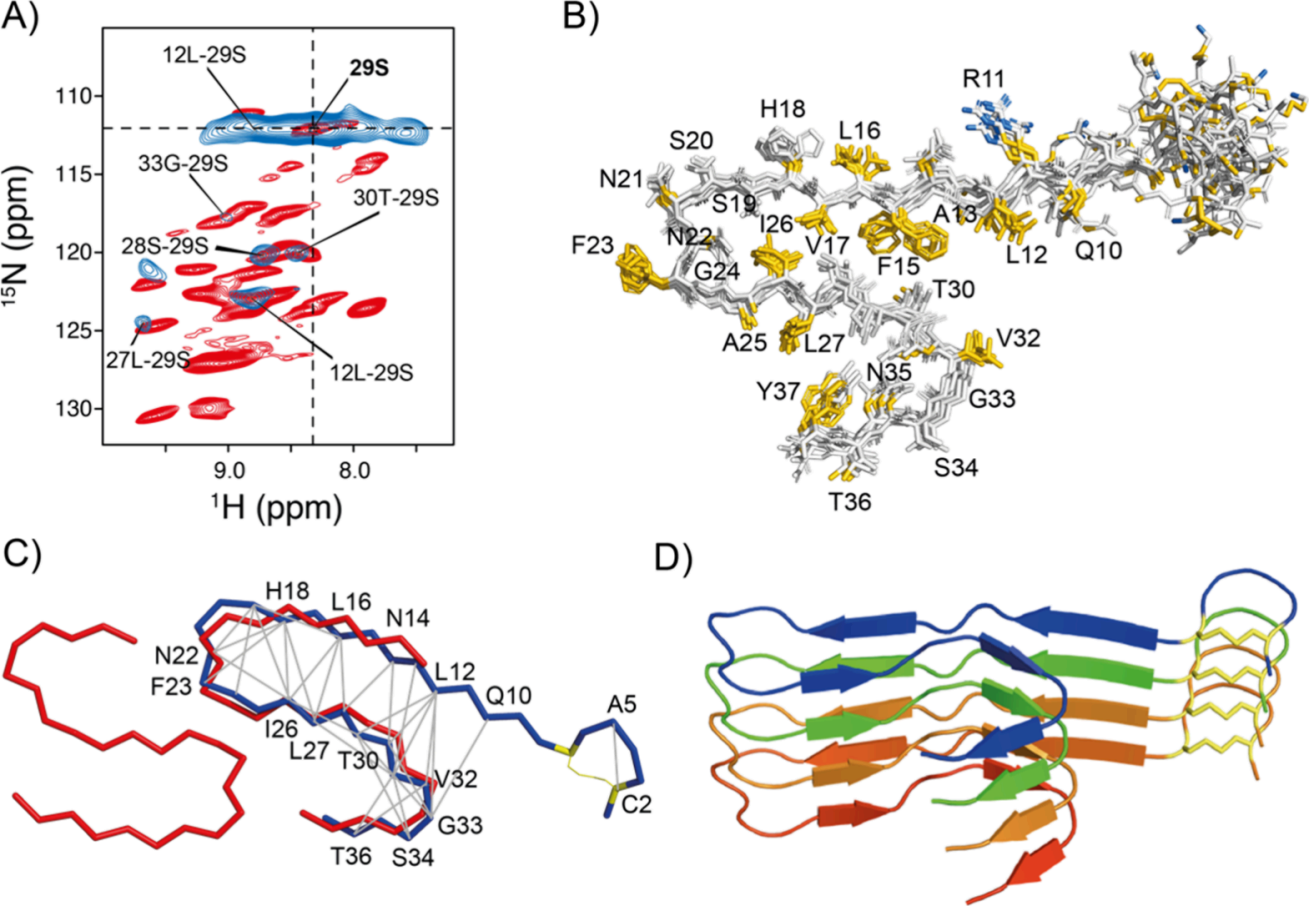
Structural analysis of hIAPP fibrils using MAS solid-state NMR data. (A) 2D trace (F2, F3) of the 3D hNh-RFDR-hNH correlation experiment (blue). The 2D trace was taken at the ^15^N chemical shift (F1) of S29. In the experiment, the RFDR mixing time was set to 6 ms. The spectrum is superimposed with a ^1^H,^15^N correlation spectrum (red) to allow for assignment. (B) Ensemble of the 10 lowest energy structures that are consistent with structure calculations using CYANA.^41^ The average heavy atom RMSD is on the order of 2 Å. Hydrophobic and charged residues are highlighted in yellow and blue, respectively, using the protocol described by Hagemans et al.^81^ (C) NMR long-range distance restraints (gray) mapped on the final CYANA structure (blue). The calculated structure is superimposed with the cryo-EM structure (red, PDB code 6Y1A).^7^ (D) Ribbon representation of the lowest energy structures of four hIAPP molecules along the fibril axis. Individual molecules are colored following rainbow colors. All figures were generated using PyMOL, Molecular Graphics System Schrödinger, LLC.^82^

A defining structural property of amyloid fibrils is the cross-β structural motif in which β-sheets are stacked upon each other with the β-sheets oriented perpendicular to the fibril axis at a distance of 4.7 Å.^1^ Amyloid fibrils may be viewed as self-assembling, quasi-one-dimensional systems. In case the subunits are arranged in a symmetrical fashion, the homogeneous core region yields a single set of resonances in NMR experiments.^78^ To determine the basic topology of the hIAPP fibril, we recorded 3D radio-frequency-driven recoupling (RFDR) experiments that directly probed ^1^H–^1^H proximities. Figure 4A shows a representative 2D trace of the 3D hNh-RFDR-hNH correlation experiment. We obtained 30 long-range distance restraints (Table S4) that are used together with the residue specific secondary chemical shift information as input for structure calculations using the program CYANA.^41^ The corresponding restraints and structure statistics are listed in Table S5. The amyloid fibril topology that is consistent with all NMR data is represented in Figure 4B. Our NMR data are in agreement with a C_2Z_ symmetric arrangement of protomers in the fibril structure, as we observe interactions involving residues N35–N21, L27–F23, and A25–F23 that presumably reflect intermolecular contacts.

Most of the long-range ^1^H^N^–^1^H^N^ contacts reflect interactions within residues 12–36. This is presumably because this part of the fibril is more densely packed. The central core of the hIAPP fibril structure is rather hydrophobic and is formed by residues of β-sheet β2 interacting through the hydrophobic side chains of F15, L16, and V17 with the side chains A25, I26, and L27 of β-sheet β3. In addition, the β1−β4 interface induces a burial of the hydrophobic side chains L12 and V32 which allows the reduction of the amount of solvent-exposed hydrophobic groups on the surface of the fibril. The solvent-exposed surface is mostly composed of polar residues. The central hydrophobic core is likely complemented by an asparagine ladder with the side chain of N21,^7^ which contributes significantly to the stability of the amyloid fibril.^79,80^ Site-directed mutagenesis studies that aimed to delineate the role of asparagine amide side chains in the sequence revealed that single-point mutations such as N21L (and not N22L) significantly disrupt the fibrillogenesis of hIAPP.^80^ Interestingly, we observe distinct Cβ chemical shifts for N21 supporting its unique role in the formation of ordered fibrous states of hIAPP (Figure S4). Glycine residues are often key and facilitate folding of an amyloid fibril structure.^44^ Often, glycines determine the end points of a β-strand and enable the formation of narrow turns. Our structure calculation reveals that restricting G24 to a β-sheet conformation results in violation of both torsional angle restraints and hydrogen bonding along the fibril axis. We observe large negative secondary chemical shifts for the central 22-NNFGAIL-27 segment, which is in agreement with previous ^13^C-detected solid-state NMR experiments.^67^ We speculate that these large negative secondary chemical shifts not only are presumably due to formation of an extended β-sheet but also reflect the strong stabilization of this region due to dimerization.^7^ This hypothesis in agreement with a X-ray crystal structure of the short hIAPP segment 22-NNFGAIL-27 suggesting that this region is not capable of forming a typical steric zipper arrangement.^75^ By contrast, a pronounced bend in the backbone facilitated by the glycine residue allows the side chain of Asn to turn inward and form a hydrogen bond with the glycine backbone carbonyl. Interestingly, this fragment structure which mainly includes residues of the connecting loop superimposes well with our NMR structural model (Figure S5). Earlier models of hIAPP fibrils suggested an extended β-sheet, including G33 (Figure 3E). However, our secondary structural analysis and the structural model imply that glycine at position 33 acts as a β-sheet disrupter and this way enables the formation of the S-shaped fibril topology. This is agreement with the recent cryo-EM amyloid structural models. We do not observe any resolved resonance for Y37. Low intensity cross-peaks for Y37 with random coil secondary chemical shifts were identified using ^13^C detected solid-state experiments indicating that this residue is not part of a β-sheet.^67^ In both cryo-EM structural models that have been solved by Röder et al.^7^ and Gallardo et al.,^8^ Y37 is part of the amyloid core and important for the S-shaped dimer formation and stabilization.^7,8^ Fluorescence quenching experiments have shown as well that the C-terminus is rigidly packed in a well-defined environment in the fiber state.^59^ We speculate that conformational heterogeneity and a differential protomer packing arrangement in the hIAPP dimer at the C-terminus results in a reduction of the peak intensity and thus in a disappearance of the resonances.

Dilution of the proton spin system by deuteration limits the number of ^1^H–^1^H contacts in general and complicates structure calculations since side chain–side chain interactions are largely missing. In addition, the distinction of inter- and intramolecular contacts in ^1^H-detected SSNMR remains a challenge for sensitivity reasons.^43^ We therefore compared our solid-state NMR structural model with the currently available cryo-EM structures. We find the best agreement between our structural model and the cryo-EM structure by Schröder and co-workers.^7^ This fibril structure satisfies all of our experimental solid-state NMR-derived distance restraints. A superimposition with the cryo-EM structure is represented in Figure 4C. We therefore employed the PDB code 6Y1A in the following as a starting point for further MD simulations to obtain a more detailed understanding of the structural differences between wt hIAPP and IAPP_C2S/C7S_ fibrils. In the MD simulations, we accounted for the solid-state NMR data and restrained distances between donor–acceptor pairs for the amino acids in the N-terminus involved in β-sheet structures according to the experimental observations. To mimic a fibril structure, eight layers of peptide were stacked upon one another and a trajectory was calculated for 1 μs for each the wildtype and the hIAPP_C2S,C7S_ variant structure. The obtained results were analyzed further only for the last 500 ns of the trajectory and the inner 4 peptide layers of the fibril calculation. Figure 3F shows the β- sheet propensities for wt hIAPP and IAPP_C2S,C7S_ fibrils as obtained from the MD simulations. We find that wt hIAPP and IAPP_C2S,C7S_ fibrils almost share identical cross-β arrangements in the central and C-terminal region. At the same time, the β-sheet at the N-terminus of IAPP_C2S/C7S_ is further extended. The N-terminal β-sheet in wt hIAPP fibrils is interrupted by a two-residue turn at positions 12 and 13. For these residues, the torsional angle restraints obtained in simulations are in full agreement with the Talos predicted experimental secondary structures (Figure 3A,E).

**Figure 5:**
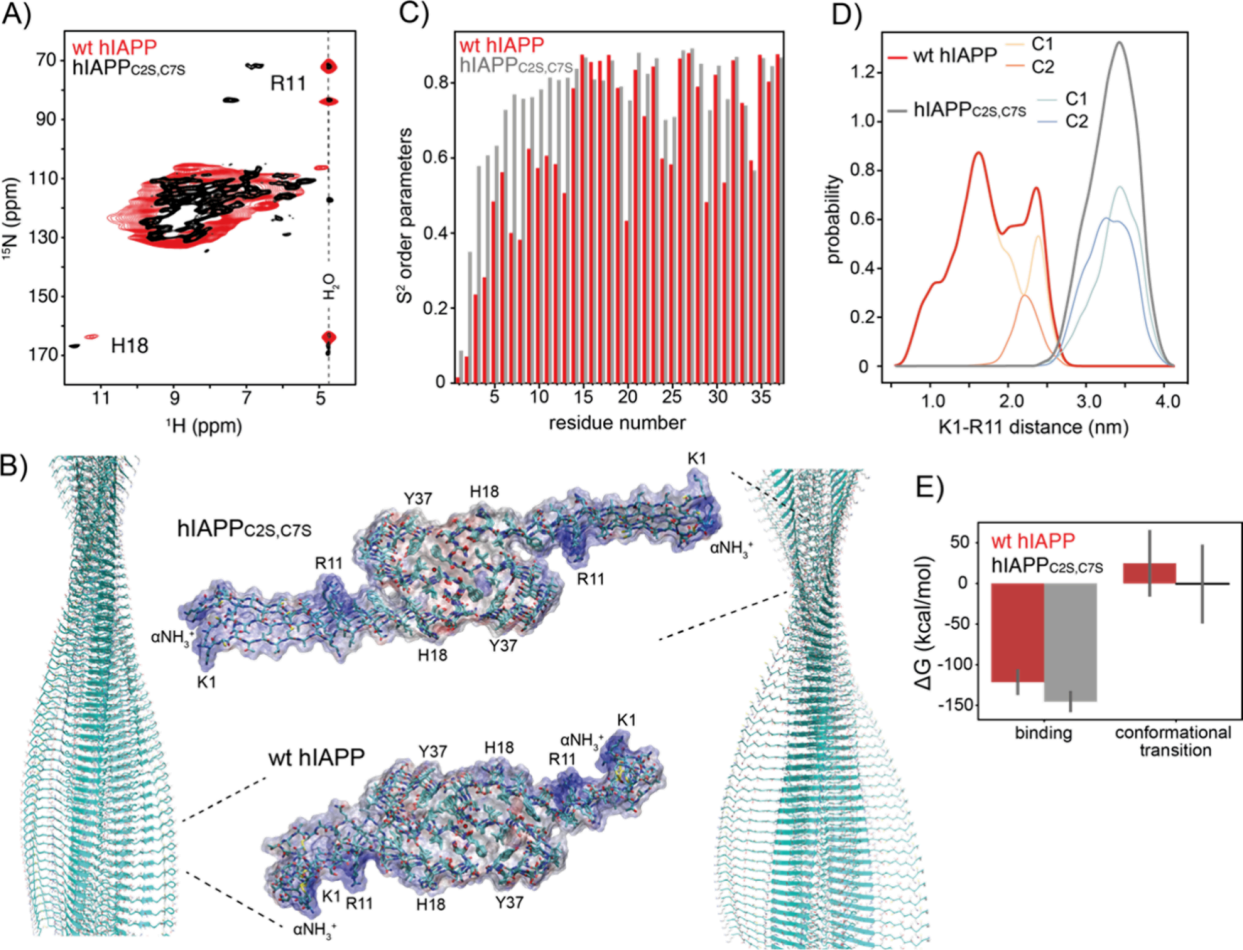
Structural analysis of the hIAPP fibril N-terminus. (A) ^1^H,^15^N correlation spectrum of wt hIAPP (red) and hIAPP_C2S,C7S_ fibrils (black). Cross peaks at the water chemical shift indicate the exchange of magnetization with the solvent. Only for hIAPP_C2S,C7S_ fibrils, the side chain resonances of R11 yield a cross peak at a proton chemical shift that is distinct from the water resonance, indicating that this residue is protected from exchange. For wt hIAPP fibrils, R11 is solvent accessible. In both peptide fibrils, the imidazole of the H18 proton is protected from exchange and in a similar chemical environment. (B) Structural representation of wt hIAPP (red) and hIAPP_C2S,C7S_ fibrils from MD simulation. For each simulation, the chain conformations from the MD trajectory that is closest to the centroid of the predominat cluster are represented. The reduced variant hIAPP_C2S,C7S_ yields a more extended structure in comparison to the oxidized counterpart with R11 pointing toward and contacting the fibril core structure. In the inset, four peptide chains extracted along the fibril axes are shown for wt hIAPP (red) and hIAPP_C2S,C7S_. Color coding is done according to electrostatic potential (blue, positively charged; red, negatively charged; white, apolar). In the hIAPP wild-type fibrils, the positive charges of the N-terminus are locally concentrated which potentially hampers association of hIAPP peptides during secondary nucleation. (C) Calculated *S*^2^ order parameter from the MD trajectory. The N-terminus of the hIAPP_C2S/C7S_ fibril chains (gray) is better ordered in comparison to the N-terminus of the wt hIAPP fibrils (red). (D) Distance between the centers of mass for the side chains of K1 and R11 for wt hIAPP (red) and hIAPP_C2S,C7S_ (gray) fibrils obtained from the MD trajectories. Distances are represented for the two predominantly populated clusters, cluster 1 (C1) and cluster 2 (C2). (E) MM/GBSA calculations for wt hIAPP (red) and hIAPP_C2S,C7S_ (gray) fibrils. The simulations indicate that the reduced peptide variant binds monomeric peptide more stably, suggesting that elongation is facilitated in comparison to the wild-type protein (left). Similar, conformational conversion from the monomeric oxidized peptide to the fibril state is energetically unfavored.

To yield qualitative information about the conformation of the N-terminus in wt hIAPP and hIAPP_C2S/C7S_ fibrils, in particular to probe solvent accessibility by NMR, we revisited the ^1^H,^15^N correlation spectra. Side chain exchangeable protons are only detectable if the respective proton is stabilized e.g. by a hydrogen bond and is thus protected from exchange.^83^ We analyzed the histidine and arginine side chain resonances, which are found in resolved spectral regions beyond the backbone amide correlation peaks (Figure 5A). For arginine, only the hIAPP_C2S,C7S_ fibril sample yields resonances with proton chemical shifts that are distinct from those of water (^15^N chemical shifts between 70 and 80 ppm). This suggests that R11 is not fully solvent accessible in hIAPP_C2S,C7S_ fibrils but partially buried and in contact with the core of the fibril structure, while R11 is fully solvent accessible in the wt hIAPP fibrils. By contrast, the histidine side chain (^15^N chemical shifts around 175 ppm) produces a single cross peak with a distinct proton chemical shift for both the wt hIAPP and the hIAPP_C2S,C7S_ fibril sample. H18 is thus protected from exchange with the solvent in both fibril structures. At the same time, the histidine side chain appears to be neutral, as a protonated imidazole ring would give rise to two proton side chain resonances. The serine and threonine hydroxyl proton exchange behavior is in agreement with the hIAPP fibril topology (Figure S6). Hydroxyl groups which are located in the amyloid core are protected from exchange (S19, S20, S28, S29, and T30), while solvent exposed side chain OH protons (T4, T6, S34, and T36) are not observable in hCH correlation experiments.^84^ The NMR spectra are in good agreement with MD simulations, with the exception of H18 and S20. In MD simulations, H18 is not involved in a hydrogen bond with S20, while the NMR spectra suggest that the side chain hydroxyl group of S20 is protected from exchange and possibly involved in a hydrogen bond with H18.

To identify and characterize representative structural features of the MD simulation, we grouped wt hIAPP and hIAPP_C2S,C7S_ fibril conformers using hierarchical RMSD clustering. The simulation was implemented by using a box that contained hIAPP dimers that are arranged in four layers. We find that both fibril structures are represented well by two clusters that are populated with 87.5/12.5% and 50%/50% for wt hIAPP and hIAPP_C2S,C7S_, respectively. The centroids of the major populated clusters are shown in Figure 5B. All clusters are represented in Figure S7. wt hIAPP clusters deviate in the orientation of the cyclic structure induced by the disulfide bond. Whereas the cyclic structure of cluster 1 is oriented in parallel to the amyloid fibril axis, cluster 2 points rather in a perpendicular direction. The hIAPP amyloid is composed of two intertwined structures of stacked hIAPP peptides. The 50%/50% symmetry obtained from the clusters of hIAPP_C2S,C7S_ is due to the rigidity of each half of the interwoven amyloid as the conformations of each of the halves span the entirety of each cluster. This leads us to believe that the rigidity of the structure does not allow us to sample diverse conformations drifting away from the initial conformation during the trajectory. However, if the simulations were allowed to run for much longer, then the two clusters may merge into a single one.

In general, the clusters for wt hIAPP and hIAPP_C2S,C7S_ are very similar; however, they differ in their packing around residues R11 and V12. We examined the distance distribution between the center of mass of residue K1 and R11 to get a better understanding of the conformational packing for the fibril N-terminus. For hIAPP_C2S,C7S_ fibrils, the distance distribution curve yields a sharp peak centered at around 3.5 nm, reflecting the repulsive interaction between the side chain of residue R11 and the core of the amyloid. By contrast, the solvated R11 in the wt hIAPP induces a more collapsed conformation as shown in Figure 5D. The MD trajectory is thus in agreement with the NMR data. The charged side chain of R11 is oriented toward the solvent for wt hIAPP. This results in a distortion and interruption of the β-sheet (Figure 5B). For IAPP_C2S,C7S_ fibrils, this is not the case. There, the side chain of R11 is only partially solvated and contacts the core of the fibril structure by pointing to the loop involving residues V32–S34. For both the wt hIAPP and variant IAPP_C2S,C7S_ fibrils, the side chains undergo significant conformational fluctuations, but the initial orientation of R11 relative to the core structure remains unchanged throughout the full MD trajectory. It should be noted that major reorientations of R11 are only possible through major changes of the peptide backbone structure that were not observed on the time scale of the MD simulations. The reduced solvent accessibility of R11 in the IAPP_C2S,C7S_ fibril variant is energetically unfavorable, but this is outbalanced by an uninterrupted β-sheet. In contrast, the wt hIAPP involves a fully solvent exposed and thus enegetically more favorable R11 side chain arrangement at the cost of distortion and interruption of the β-sheet (Figure 5B).

MD simulations show that the S^2^ order parameters in wt hIAPP fibrils are lower at the N-terminus (Figure 5C). Residues in the N-terminus of hIAPP_C2S,C7S_ are more rigid in comparison to wildtype. This is in agreement with the experimental NMR peak intensities (Figure 3D). For wt hIAPP, we observe that N-terminal hCANH peak intensities are lower in comparison to those of residues that are located in the core of the fibril. By contrast, the peak intensity distribution is very uniform for hIAPP_C2S,C7S_ fibrils. We hypothesize that the increased dynamics of the N-terminus of wt hIAPP impedes the agglomeration of hIAPP and slows secondary nucleation. At the same time, this observation suggests that the reduced variant is more favorably packed.

To yield a better understanding of the peptide–peptide interactions involved in secondary nucleation dependent seeding, we calculated the electrostatic surface potential for wt hIAPP and hIAPP_C2S,C7S_ clusters (Figure 5B). Under our experimental conditions, hIAPP contains only three charged groups, the N-terminal α-amino group and K1 and R11. hIAPP does not contain negatively charged side chains that can potentially compensate for the positive charges of the N-terminus. For wt hIAPP, the proximity of the charged N-terminus and the side chains of K1 and R11 induces an accumulation of positive charge. By contrast, the structural clusters for hIAPP_C2S,C7S_ fibrils rather feature extended structures in which the positive charges are more evenly distributed on the fibril surface. The arrangement and the packing of the hIAPP N-terminus allows us to shed light on the mechanism of secondary nucleation and elongation. In this process, monomeric or oligomeric proteins nucleate at the fibril seed surface and convert into new fibrils. The wt hIAPP fibril structure is characterized by the disulfide induced cyclic loop that implicates clustering of the three positive charges. We hypothesize that this local increase of positive charges decreases the available surface area for seeding and impedes the adherence of the monomeric peptide.

The seeded ThT aggregation kinetics of wt hIAPP suggest that fibril growth requires an activation step for seeding. Activation implies either oligomer formation or conformational rearrangement of the monomeric peptide prior to secondary nucleation. By contrast, hIAPP_C2S,C7S_ fibrils are rather extended facilitating the adherence of monomeric hIAPP peptide.

We find that activation is seed concentration dependent (Figure 1D,E). Activation is less pronounced at very high seed concentrations, suggesting that elongation contributes significantly to fibril growth. In order to energetically characterize the binding and structure formation at the fibril ends of both wt and variant hIAPP, we performed MM/GBSA calculations. To estimate the binding affinity, we compared a system with two amyloid layers with two systems containing a single layer (Figure 5E). Similarly, we compared a system with two amyloid layers with two unbound peptides adopting a helical structure (PDB code 5MGQ). MM/GBSA results were obtained as the average from 5 independent 200 ns simulations for each system. The calculations show that binding of wt hIAPP peptide to an wt hIAPP amyloid layer is energetically less favorable in comparison to binding of the reduced variant to a hIAPP_C2S,C7S_ fibril layer. At the same time, wt hIAPP amyloid structure formation starting from the helical conformation of the oxidized peptide is even less favorable. This suggests that the N-terminus of hIAPP has a significant impact on the fibril elongation event when wt hIAPP and hIAPP_C2S,C7S_ fibrils are compared.

## Conclusions

We have shown that hIAPP adopts a fibril topology that is similar to the recently determined cryo-EM structures for the core region. Previous MD simulations and advanced sampling methods^85^ concluded from the experimental cryo-EM density that the N-terminus of the hIAPP fibril structure is heterogeneous. By contrast, the NMR data yield a single set of resonances, suggesting a high degree of structural homogeneity at the N-terminus. It has been demonstrated previously that the N-terminus plays an important role in seeding and interactions with chaperones. We find that wt hIAPP and hIAPP_C2S,C7S_ fibrils have a similar fibrillar core but differ in the arrangement of the N-terminus. The disulfide bond induced cyclic loop in wt hIAPP fibrils yields a clustering of three positive charges and impedes the adherence of the monomeric peptide. By contrast, the hIAPP_C2S,C7S_ fibrils are rather extended. The positive charges are more evenly distributed on the fibril surface, which facilitates secondary nucleation and fibril growth. MD calculations indicate that packing of hIAPP_C2S,C7S_ monomers in the variant fibrils is energetically more favorable suggesting that elongation is facilitated for this type of fibrils. The structure enables a mechanistic understanding of fibril growth by elongation and secondary nucleation and sheds light on the importance of the fuzzy coat that surrounds the amyloid core for fibril formation.

## Supporting Information Available

Strip plots of 3D hCANH, hcoCAcoNH, and hcaCBcaNH experiments for wt-hIAPP fibrils and IAPP_C2S,C7S_ fibrils; superposition of ^1^H,^15^N correlation spectra obtained for fibril samples obtained by using *ex vivo* seeds and hIAPP fibrils obtained without seeds; correlation of ^13^Cα chemical shifts of wt-hIAPP fibrils and hIAPP_C2S,C7S_ fibrils for residues Q10-T36; ^13^C detected 2D CO–CA/CB correlation spectrum for Asn and Gln side chains recorded for wt hIAPP fibrils; superposition of hIAPP fibril structures obtained from X-ray crystallography and the solid-state NMR structural model; analysis of the solvent accessibility of serine and threonine side chains hydroxyl groups in wt-hIAPP fibrils; clusters analysis for wt hIAPP and hIAPP_C2S/C7S_ fibrils; kinetic analysis of ThT aggregation data for wt hIAPP and hIAPP_C2S,C7S_; chemical shift assignments for wt hIAPP and hIAPP_C2S,C7S_ fibril samples; medium and long range backbone ^1^H–^1^H distance restraints from 3D RFDR-hNhhNH spectra; restraint and structure statistics (PDF)
