# Supplementary material for: Structural Insights into Seeding Mechanisms of hIAPP Fibril Formation
Source: J Am Chem Soc. 2024 May 9;146(20):13783–96. doi: 10.1021/jacs.3c14233 (PMC11117405; doi:10.1021/jacs.3c14233)
Supplement: Supplementary file 1 — ja3c14233_si_001.pdf [file ja3c14233_si_001.pdf]

Supporting Information for the manuscript

Structural insights into seeding mechanisms of hIAPP fibril formation

Saba Suladze[1,2], Christian Sustay Martinez[3], Diana C. Rodriguez Camargo[1,2], Jonas Engler[1,2], Natalia Rodina[1,2], Riddhiman Sarkar[1,2], Martin Zacharias[3], Bernd Reif[1,2]*

[1] Bayerisches NMR Zentrum (BNMRZ) at the Department of Biosciences, School of Natural Sciences, Technische Universität München, Munich, Germany

[2] Helmholtz-Zentrum München (HMGU), Deutsches Forschungszentrum für Gesundheit und Umwelt, Institute of Structural Biology (STB), Ingolstädter Landstr. 1, 85764 Neuherberg, Germany

[3] Center for Functional Protein Assemblies (CPA), Department of Bioscience, TUM School of Natural Sciences, Technische Universität München, Ernst-Otto-Fischer-Straße 8, 85747 Garching, Germany

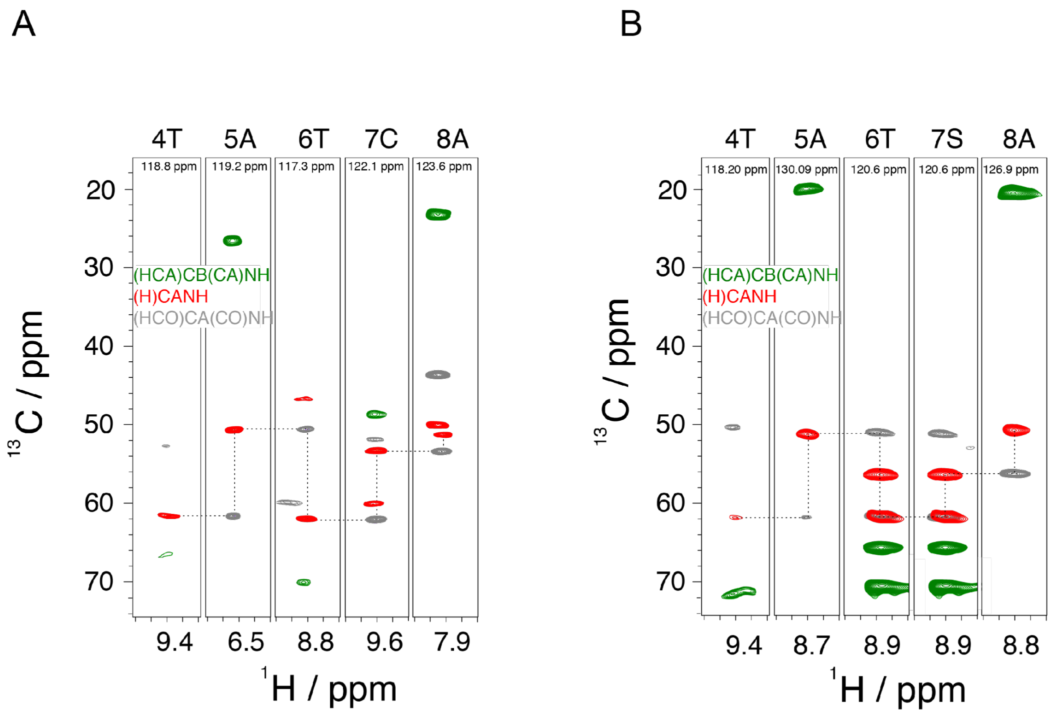

Figure S1. Strip plot of the proton-detected 3D hCANH (red), hcoCAcoNH (grey) and hcaCBcaNH (green) experiments, yielding the sequential walk between residues 4 and 8 for wt-hIAPP fibrils (A) and the disulfide-free (B) $IAPP_{C2S,C7S}$ fibril variant.

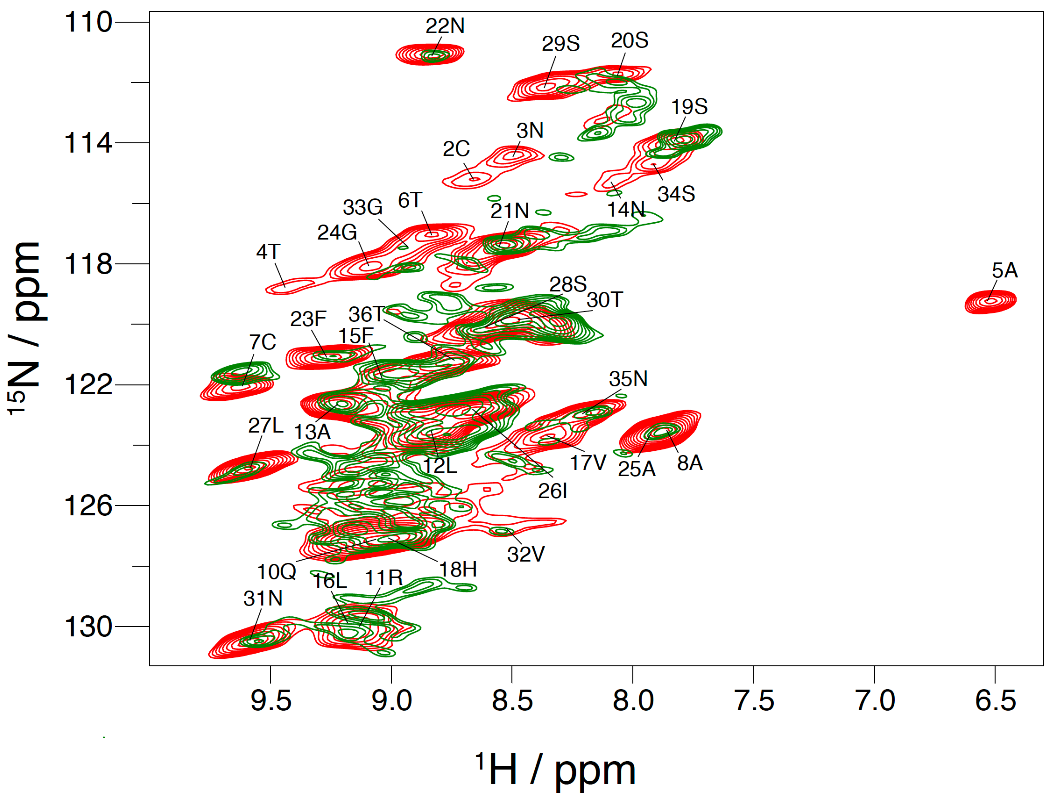

Figure S2. Overlay of ^{1}H,^{15}N correlation spectra obtained from fibril samples obtained by seeding using *ex vivo* seeds extracted from pancreatic islets of hIAPP transgenic mice[1] (green) and hiapp fibrils obtained without seeds (red).

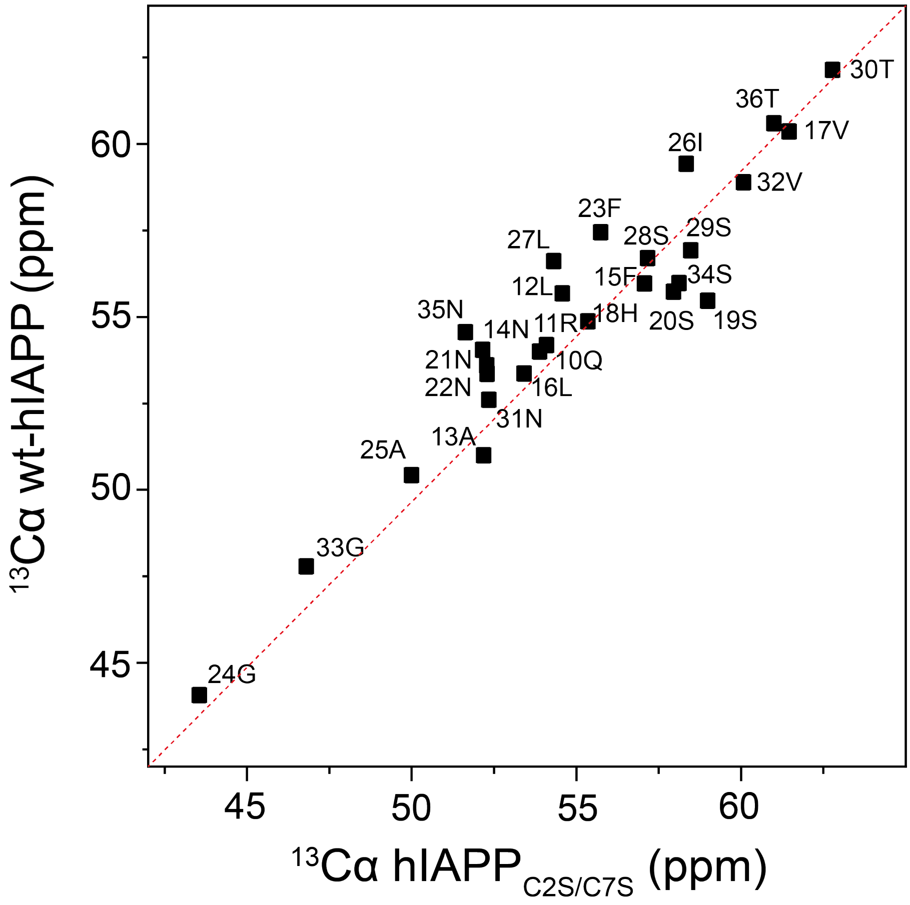

Figure S3. Correlation of $^{13}C\alpha$ chemical shifts of wt-hIAPP fibrils (vertical axis) and $hIAPP_{C2S,C7S}$ fibrils for residues Q10-T36. The high correlation coefficient suggests that the backbone structures for the two preparations are highly similar.

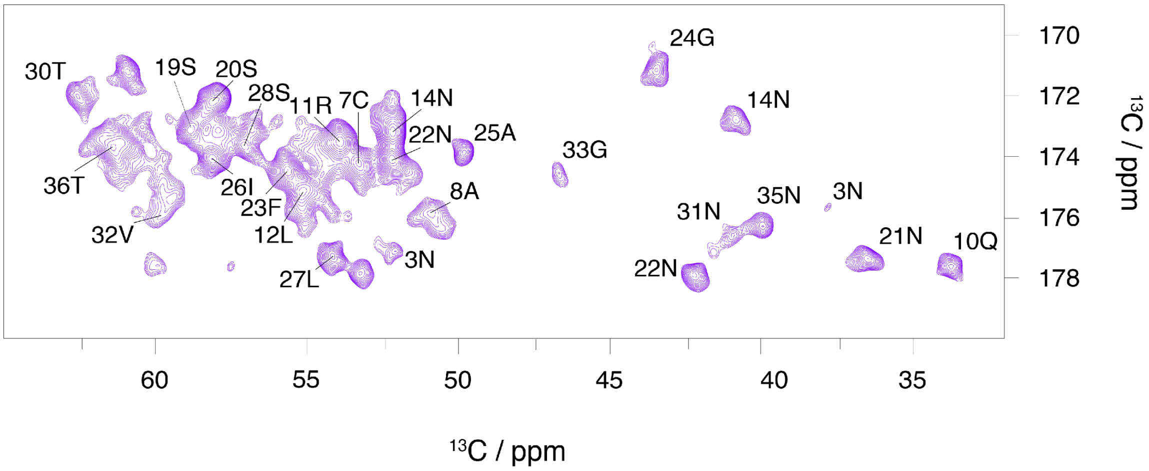

Figure S4. 2D ^{13}C detected CO–CA/CB correlation spectrum for wt hIAPP fibrils illustrating the distinct local environments for N21 in comparison to the neighboring N22 as judged by the very distinct ^{13}Cβ chemical shifts.

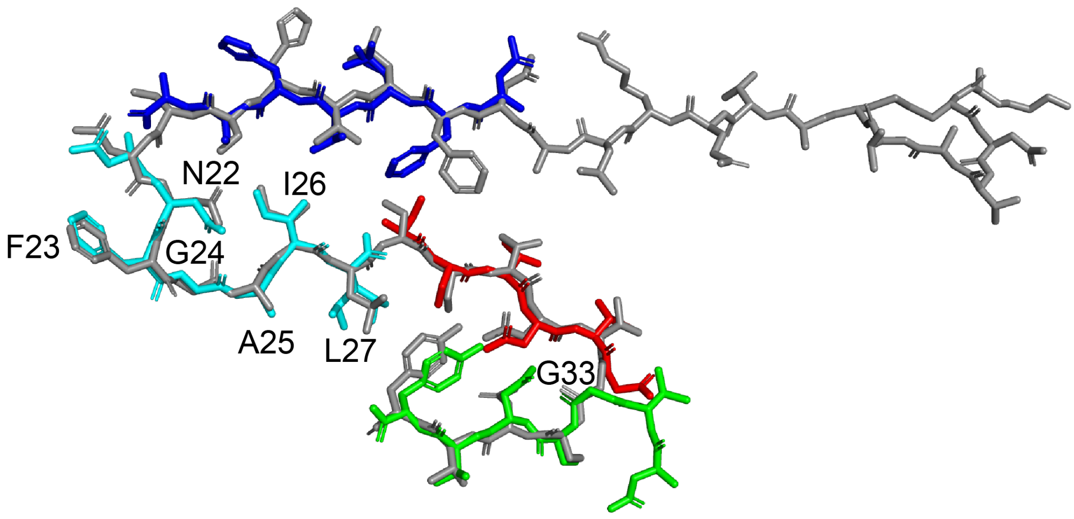

Figure S5. Superposition of hIAPP fibril structures obtained from X-ray crystallography (aa 14-20, PDB: 3FTH, blue; aa 21-27, PDB: 3DGJ, cyan; aa 28-33, PDB: 3FTR, red; aa 31-37, PDB: 3FTL, green),[2,3] and the solid-state NMR structural model (grey).

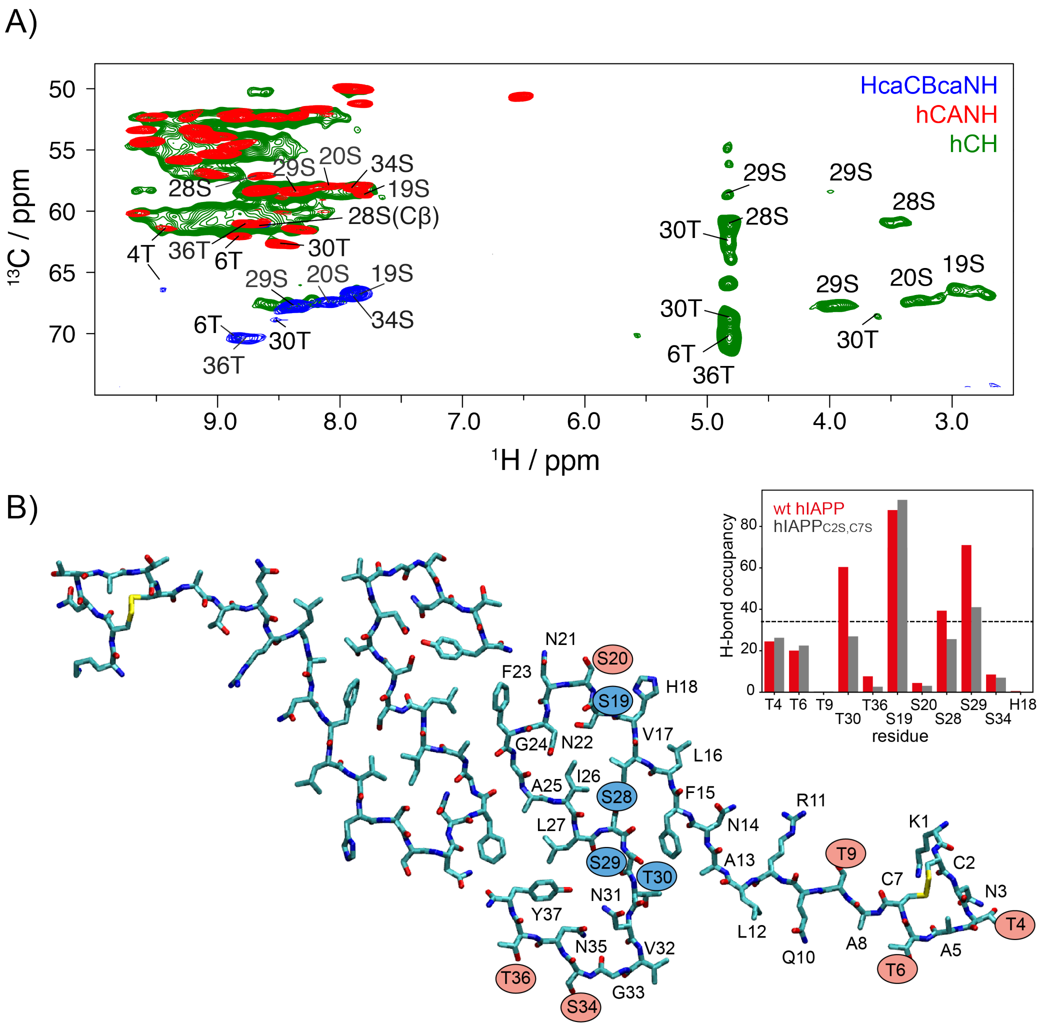

Figure S6. (A) Superposition of hCH (green), hCANH (red) and HcaCBcaNH (blue) correlation spectra that yield the assignment of serine and threonine side chain OH chemical shifts in wt hIAPP fibrils. (B) Cross section through a wt hIAPP amyloid fibril. Serine and threonine side chains are color coded according to their hydroxyl proton exchange behaviour which is indicative for hydrogen bonding. Red represents fast exchanging hydroxyl protons, while blue highlights OH groups involved in a hydrogen bond. The likelihood for a serine and threonine side chain to be involved in a hydrogen bond (as derived from the molecular dynamics trajectories for wt hIAPP and $hIAPP_{C2S,C7S}$ fibrils) is indicated in the inset. In the MD simulations, H18 is not involved in a hydrogen bond with S20, while the NMR spectra suggest that the side chain hydroxyl group of S20 is protected from exchange and possibly involved in a hydrogen bond with H18.

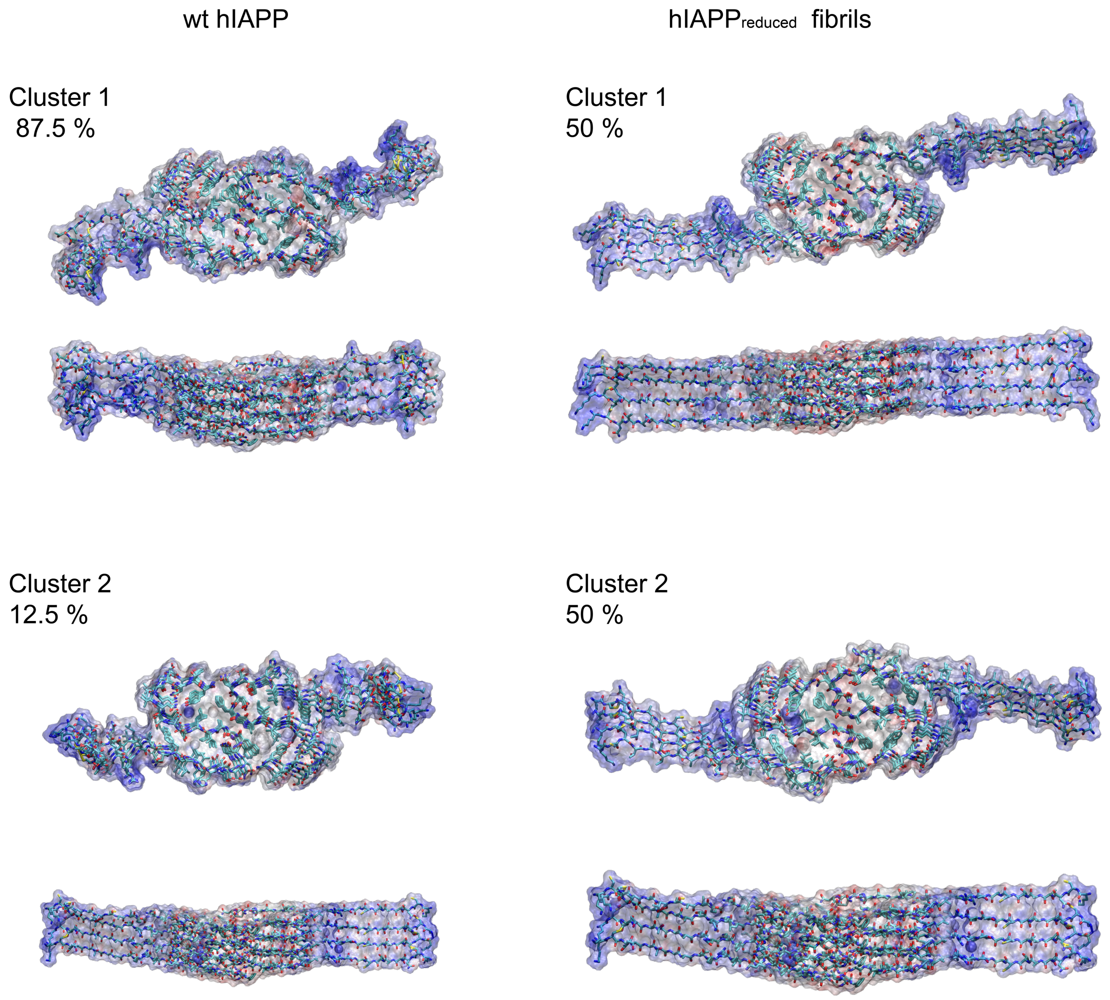

Figure S7. Clusters analysis for wt hIAPP and hIAPP$_{C2S/C7S}$ fibrils. Using hierarchical RMSD clustering, the fibril conformers are grouped into clusters. We find that both fibril structures are represented well by two clusters that are populated with 87.5/ 12.5% and 50%/ 50% for wt hIAPP and hIAPP$_{C2S,C7S}$, respectively.

Table S1: Kinetic parameters for global fitting of the experimental data for wt hIAPP and $hIAPP_{C2S,C7S}$ to a secondary nucleation dominated model, where $k+$, k_n and k_2 are the elongation rate constant, primary nucleation rate constant and secondary nucleation rate constant, respectively. Platform generates combined rate constants for primary nucleation k_n x $k+$ and secondary nucleation k_2 x $k+$ pathways.

	wt hIAPP	$hIAPP_{C2S,C7S}$
k_n x $k+$ (primary)	1.1×10^{7}	1.6×10^{9}
k_2 x $k+$ (secondary)	3.2×10^{16}	4.5×10^{15}

Table S2: Chemical shifts in ppm. Chemical shifts in ppm derived from wt hIAPP fibril sample and used for secondary chemical shift calculation. Small letters for cysteine residues indicate the oxidized disulfide bridge.

S2 Table, Chemical shifts in ppm						
	residue	H	N	Cα	Cβ	CO
1	K			55.22		179.40
2	c	8.67	115.24	59.05		173.68
3	N	8.50	114.41	52.44	37.87	177.15
4	T	9.44	118.81	61.50	66.59	174.69
5	A	6.53	119.19	50.62	26.42	176.29
6	T	8.84	117.27	62.11	70.11	173.58
7	c	9.62	121.99	53.36	48.67	174.20
8	A	7.84	123.62	51.20	22.99	
9	T			61.09		171.46
10	Q	9.04	126.98	53.88	34.46	173.37
11	R	9.10	130.10	54.09	32.96	173.39
12	L	8.84	123.49	54.57	43.04	175.38
13	A	9.24	122.58	52.19	16.34	175.10
14	N	8.10	115.36	52.15	40.77	173.31
15	F	9.03	122.49	57.07	41.38	173.31
16	L	9.17	130.17	53.41	44.96	174.66
17	V	8.34	123.66	61.46	33.48	171.38
18	H	8.98	127.03	55.35	39.60	175.79
19	S	7.82	113.78	58.99	66.68	173.13
20	S	8.08	111.75	57.95	67.39	172.08
21	N	8.55	117.42	52.28	36.63	173.13
22	N	8.84	111.05	52.29	42.27	174.13
23	F	9.27	121.09	55.74	41.35	174.48
24	G	9.08	118.06	43.55		171.27
25	A	7.92	123.70	50.00	23.03	173.88
26	I	8.65	122.82	58.34	36.61	174.11
27	L	9.59	124.68	54.31	44.74	177.42
28	S	8.66	120.13	57.16	61.10	173.83
29	S	8.36	112.10	58.47	67.71	172.41
30	T	8.48	119.94	62.78	68.87	172.07
31	N	9.60	130.43	52.34	40.90	174.18
32	V	8.47	126.73	60.08	34.40	176.18
33	G	8.98	117.89	46.80		174.82
34	S	7.91	114.76	58.12	66.74	173.27
35	N	8.19	123.00	51.63	40.23	174.42
36	T	8.78	121.23	61.00	70.32	
37	Y					

Table S3: Chemical shifts in ppm. Chemical shifts in ppm derived from hIAPP$_{C2S,C7S}$ fibril sample and used for secondary chemical shift calculation.

	residue	H	N	Cα	Cβ	CO
1	K					
2	S					
3	N			50.52		174.09
4	T	8.73	118.12	61.77	71.39	172.51
5	A	8.76	130.14	51.12	20.24	175.80
6	T	8.94	120.80	61.55	70.35	173.03
7	S	8.92	120.50	56.31	65.52	171.83
8	A	8.79	127.11	50.48	20.54	175.95
9	T	9.31	122.99	60.79	70.68	170.73
10	Q	9.21	128.50	54.00	33.41	174.22
11	R	9.02	126.16	54.19	33.59	171.49
12	L	8.25	122.09	55.68	42.52	175.60
13	A	8.94	123.33	51.00	22.53	176.37
14	N	8.92	115.68	54.05	34.59	173.45
15	F	9.21	124.56	55.97	43.74	171.87
16	L	9.10	122.27	53.37	42.42	173.57
17	V	9.45	123.78	60.37	34.29	172.75
18	H	9.25	127.06	54.88	31.78	173.82
19	S	9.33	116.13	55.47	63.41	173.76
20	S	9.07	116.09	55.73	63.84	174.15
21	N	8.69	121.35	53.60	40.18	171.89
22	N	8.15	117.78	53.35	39.77	172.43
23	F	9.86	123.80	57.45	40.45	174.16
24	G	7.12	108.77	44.07		171.47
25	A	8.88	128.47	50.43	20.76	175.88
26	I	8.92	119.30	59.43	41.13	173.51
27	L	9.00	124.69	56.62	44.01	
28	S			56.70		174.95
29	S	8.24	120.58	56.93	61.87	172.75
30	T	9.73	125.50	62.15	71.26	170.73
31	N	9.38	128.92	52.61	41.02	172.44
32	V	8.94	123.42	58.90	35.22	174.77
33	G	9.28	113.82	47.79		170.70
34	S	8.31	112.08	55.98	66.16	174.04
35	N	8.63	125.56	54.56	43.16	173.95
36	T	9.37	123.52	60.60	70.61	
37	Y					

Table S4: Manually assigned backbone ^{1}H-^{1}H medium and long-range restraints for the structure calculation. The restraints have been collected from the 6 ms 3D RFDR-NhhNH spectra. Only medium-range contacts that satisfy $i - j = 3$ are listed here.

	ω1- ω2	Distance in NMR bundle in Å	Distance in CryoEM structure
1	2 CYS-5 ALA	10.1	
2	10 GLN-33 GLY	12.0	
3	11 ARG-31 ASN	11.5	
4	12 LEU-29 SER	11.9	
5	12 LEU-30 THR	9.7	
6	12 LEU-31 ASN	9.2	
7	12 LEU-32 VAL	10.5	
8	12 LEU-33 GLY	12.0	
9	13 ALA-31 ASN	10.1	12.3
10	14 ASN-28 SER	9.6	12.4
11	14 ASN-30 THR	9.0	10.1
12	16 LEU-19 SER	10.3	9.9
13	16 LEU-26 ILE	12.0	13.3
14	16 LEU-27 LEU	10.3	12.3
15	16 LEU-28 SER	9.7	10.0
16	18 HIS-22 ASN	9.0	10.3
17	18 HIS-26 ILE	9.7	10.8
18	18 HIS-27 LEU	10.4	11.2
19	19 SER-22 ASN	6.9	7.4
20	19 SER-24 GLY	9.7	11.1
21	19 SER-26 ILE	11.0	10.4
22	22 ASN-26 ILE	9.0	8.4
23	26 ILE-29 SER	9.5	10.1
24	27 LEU-31 ASN	12.0	12.3
25	29 SER-33 GLY	12.0	11.3
26	30 THR-33 GLY	10.0	8.1
27	30 THR-34 SER	11.5	11.8
28	31 ASN-34 SER	8.7	9.0
29	31 ASN-36 THR	9.7	9.9
30	32 VAL-36 THR	9.2	10.0

Table S5. Restraint and structure statistics. [a]Each group of symmetrically equivalent distance restraints is counted as a single restraint. [b]Each hydrogen bond was restrained by two upper and two lower distance bounds ($d_{H..O}$ and $d_{N..O}$). [c]The average value and the standard deviation over the 10 conformers that represent the NMR structure are given. [d]Close contacts are considered within 1.6 Å for H atoms, 2.2 Å for heavy atoms.[e]Residues with sum of phi and psi order parameters > 1.8.[41]

**Conformational restraints (per monomer):[a]**	
Distance restraints from solid state NMR spectra:[a]	66
sequential ($\|i-j\| = 1$)	26
medium range ($2 \leq \|i-j\| \leq 3$)	16
long range ($\|i-j\| \geq 5$)	24
Restrained hydrogen bonds[b]	17
Dihedral angle restraints (ϕ/ψ)	38
**Restraint violations:[c]**	**(mean±sd)**
CYANA target function value (Å^2)	2.06 ± 0.35
RMS distance restraint violation (Å)	0.024 ± 0.002
RMS dihedral angle restraint violation (°)	1.014 ± 0.110
**Deviations from Ideal Geometry**	
Number of close contacts[d]	0
RMS deviation for bond angles (°)	0.2
RMS deviation for bond lengths (Å)	0.001
**RMSD from average structure:**	
**All residues (Å)**<br>Backbone atoms	1.5
Heavy atoms of all residues	1.9
**Ordered residues (Å)[e]**<br>Backbone atoms	0.8
Heavy atoms	1.2

References

1. Franko, A., Rodriguez Camargo, D. C., Böddrich, A., Garg, D., Rodriguez Camargo, A., Rozman, J., Rathkolb, B., Janik, D., Aichler, M., Feuchtinger, A., Neff, F., Fuchs, H., Wanker, E. E., Reif, B., Häring, H.-U., Peter, A. & Hrabě de Angelis, M. Epigallocatechin gallate (EGCG) reduces the intensity of pancreatic amyloid fibrils in human islet amyloid polypeptide (hIAPP) transgenic mice. Sci. Rep. 8, e1116 (2018).
2. Wiltzius, J. J. W., Sievers, S. A., Sawaya, M. R., Cascio, D., Popov, D., Riekel, C. & Eisenberg, D. Atomic structure of the cross-beta spine of islet amyloid polypeptide (amylin). Prot. Sci. 17, 1467-1474 (2008).
3. Wiltzius, J. J. W., Landau, M., Nelson, R., Sawaya, M. R., Apostol, M. I., Goldschmidt, L., Soriaga, A. B., Cascio, D., Rajashankar, K. & Eisenberg, D. Molecular mechanisms for protein-encoded inheritance. Nat. Struct. Mol. Biol. 16, 973-979 (2009).
